# *Preventing Chronic Disease* Collection: From Data to Action: National, State, and Local Efforts to End Menthol and Other Flavored Commercial Tobacco Product Use

**DOI:** 10.5888/pcd21.240143

**Published:** 2024-05-30

**Authors:** LaTisha L. Marshall, Leslie Norman, Shyanika W. Rose, Tung-Sung Tseng

**Affiliations:** 1 Office on Smoking and Health, National Center for Chronic Disease Prevention and Health Promotion, Centers for Disease Control and Prevention, Atlanta, Georgia; 2 University of Kentucky, College of Medicine, Behavioral Science and Center for Health Equity Transformation, Lexington, Kentucky; 3 Louisiana State University Health Sciences Center, Behavioral and Community Health Sciences, School of Public Health, New Orleans, Louisiana

The use of menthol and other flavored commercial tobacco products poses a serious risk to public health, and its elimination is critical to achieving health equity (1). Targeted marketing of these products in specific populations (2,3) has contributed to health inequities through increased likelihood of initiation (3–5) and continued use and decreased successful cessation (4,6). Disparities related to its use exist across and within populations (7,8). Activities at the national, state, and local levels can help end the use of menthol and flavored tobacco products and reduce their overall tobacco-related health burden.

This *Preventing Chronic Disease* collection features 9 articles that enhance our understanding of public health’s role in reducing tobacco-related diseases and deaths, highlight menthol and other flavored tobacco surveillance data, and provide examples of state and local activities implemented in this area.

The first article, by Marshall and colleagues (9), describes the Centers for Disease Control and Prevention’s (CDC’s) National and State Tobacco Control Program (NTCP) and its role in reducing chronic disease illness, death, and disability related to commercial tobacco use and dependence and secondhand smoke exposure in the US. The NTCP supports evidence-based policy, systems, and environmental strategies (PSEs) as outlined in CDC’s *Best Practices for Comprehensive Tobacco Control Programs* (10) to address its 4 goals: 1) prevent initiation of commercial tobacco product use (including emerging products and e-cigarettes) among youth and young adults, 2) promote quitting among adults and youth, 3) eliminate exposure to secondhand smoke, and 4) identify and eliminate tobacco-related disparities among population groups. (This goal has since been updated to the following: Advance health equity by identifying and eliminating commercial tobacco product–related inequities and disparities.) NTCP disseminates the best available evidence for interventions that work to achieve its 4 goals, facilitates strategic partnerships and community engagement, and leverages internal and external resources important in addressing menthol and other flavored tobacco product use. NTCP also supports activities that are reflected in the themes of the 8 remaining articles in the collection:

- Prevalence, trends, and disparities among youth and adults
- Community engagement and social media campaigns
- Lessons learned from policy implementation

## Prevalence, Trends, and Disparities Among Youth and Adults

Surveillance of tobacco use patterns among youth and adults is a key theme in this collection. Cornelius and colleagues (11) examined the prevalence of menthol-flavored tobacco use among US middle and high school students. Among all students who reported current use of any tobacco product in 2022, approximately 24% reported using a menthol-flavored tobacco product (11). Their findings show that prevalence was highest among high school students (24.3%) and males (25.6%) (11). Among racial and ethnic groups, the prevalence was highest among non-Hispanic White students (30.1%) and lowest among non-Hispanic Black students (7.8%) (11). This result contrasts with earlier findings on menthol-flavored cigarette use among youth and adults (12,13); however, a recent study on cigarette smoking among youth reported similar findings (14). Cornelius and colleagues (11) acknowledge this may be associated with non-Hispanic Black youth starting to smoke at a later age (15,16) and a lower prevalence of smoking among youth (17,18). The article did not address other forms of combustible smoking, such as cigar use.

Cheng and colleagues (19) reported a significant increase in the prevalence of menthol cigarette use among adults aged 20 years or older who smoke, from 22.9% (1999–2002) to 35.9% (2015–2018). Non-Hispanic Black adults who smoke had the highest overall prevalence of menthol cigarette use (73.0%) (19). The highest increase occurred among Mexican American adults, from 12.8% to 31.0%, and adults with fair or poor health status, from 21.8% to 37.0% (19).

This collection does not address cessation among adults who use menthol-flavored tobacco products. Cornelius et al found that the percentage of adults aged 18 years or older who smoked and were interested in quitting ranged from 68.2% in Alabama to 87.5% in Connecticut in 2018–2019 (20). Past year quit attempts ranged from 44.1% in Tennessee to 62.8% in Rhode Island (20). Several states with the highest smoking prevalence reported the lowest prevalence of interest in quitting, quit attempts, receipt of advice to quit, and use of counseling and/or medication (20). These findings do provide evidence that most adults who smoke would like to quit (20). We do not know if this is true among adults who smoke menthol-flavored cigarettes.

## Community Engagement and Social Media Campaigns

Community engagement and social media campaign approaches to address menthol cigarette use and prevention are key themes in this collection. Caldwell and colleagues (21) developed a Community Capacity Building Curriculum to operationalize the foundational framework of the Community Development Model (22). This model prioritizes community members’ lived experiences, encouraging them to identify their unique needs and assets to achieve their desired policy, systems, and environmental changes.

Social media is also an effective approach for disease prevention and health promotion (23). Eggers and colleagues (24) evaluated a New York media campaign developed collaboratively with community partners. This study aimed to assess campaign awareness, audience reactions, and campaign-related attitudes and behaviors among community members aged 18 years or older. They suggest that community education campaigns can play an important role in raising awareness of the impact of menthol tobacco products in Black communities and help build public support for local menthol restrictions.

To address local disparities in menthol cigarette use and to support a recently adopted flavor ban in Los Angeles County, Humphrey and colleagues (25) surveyed 2 groups of people aged 18 years or older (public health professionals and people who are current smokers, are former smokers, or live with a current smoker of menthol cigarettes) to describe how a local health department used appealing creative materials and messaging reminiscent of tobacco marketing tactics to develop a health marketing campaign called “Done with Menthol.” The results of the survey were used to inform the development of this campaign. After the campaign’s initial run, the quitline call volumes for African American and Latino subgroups were 1.9 and 1.8 times higher, respectively, than the average inbound call volume for corresponding months during 2018–2019 (25). This media campaign resulted in over 66 million impressions and offered free or low-cost, accessible resources to county residents interested in tobacco use cessation. Their study supports previous findings that social media can influence hard-to-reach populations to improve health outcomes (26).

## Lessons Learned From Policy Implementation

Understanding the factors supporting or impeding policy change is another key theme in this collection. Hellesen and colleagues (27) evaluated data from 36 local grantees of the California Tobacco Prevention Program who worked to prohibit the sale of flavored tobacco products in their respective jurisdictions. Over half of these grantees spoke with community decision makers between 2017 and 2021. Their work resulted in the passage of new flavor policies in 19 local jurisdictions covered by the grantees (27). The authors reported that some factors contributing to a policy change include youth involvement, demonstrating need and public support for a ban, identifying a champion, and involving a community coalition.

Guglielmo and colleagues (28) examined additional approaches to support flavor policy adoption by 86 local communities in Los Angeles County. They found that areas with a community engagement campaign on flavor bans were more likely to pass the policy, as were those with prior experience with adopting other tobacco control ordinances, such as smokefree multi-unit housing, and those with neighboring jurisdictions that had already passed a tobacco retailer licensing policy (28). This finding suggests that local communities can ready themselves for flavor policy passage by implementing related tobacco control policies and conducting targeted community-engagement campaigns.

Caldwell and colleagues (21) described the Community Capacity Building Curriculum developed by the Center for Black Health & Equity, as theory-based, practical, and strategic guidance for community coalitions and advocacy groups to build community mobilization and menthol and flavor policy adoption in Black communities and other communities of color. This curriculum centers on health equity and social justice through multiethnic, multigenerational coalitions of partners. The curriculum has resulted in beneficial policy changes in several communities through a strong community-led process and serves as a valuable model for communities experiencing tobacco-related disparities.

## Actions to Curb the Use of Menthol and Other Flavored Tobacco Products

Although progress has been made in reducing cigarette smoking overall (29), the use of menthol cigarettes has increased and may contribute to disparities observed among subpopulations (8). Furthermore, the focused marketing of menthol and other flavored tobacco products highlights the structural barriers and unjust practices that are intentionally aimed at subsets of the US population, such as Black communities (30).

Despite differences in capacity, funding, and experience across states and localities, more than 300 local jurisdictions and 2 states have restricted the sale of menthol and other flavored tobacco products of various types (31). The US Food and Drug Administration (FDA), the agency responsible for regulating tobacco products in the US, issued 2 proposed rules in April 2022 to prohibit menthol as a characterizing flavor in cigarettes and all characterizing flavors in cigars (32). Final rules are pending (33,34).

Sharing evidence about tobacco interventions that work (10) can help states, tribes, localities, and communities mobilize partners to promote and implement equitable policies and resolutions, systems and environmental changes that can prevent tobacco initiation and support individuals who are ready to quit. Cessation support should be facilitated by engaging with community members to understand their needs to create and implement culturally appropriate interventions that resonate with the community of focus.

This collection shows public health’s role in educating communities about evidence-based interventions, including policies, to create healthier and more equitable communities, particularly among those who have been burdened by menthol and flavored tobacco.
